# Supplementary material for: Machine-Learning-Based Classification Model to Address Diagnostic Challenges in Transbronchial Lung Biopsy
Source: Cancers (Basel). 2024 Feb 9;16(4):731. doi: 10.3390/cancers16040731 (PMC10886691; doi:10.3390/cancers16040731)
Supplement: Supplementary file 1 [file cancers-16-00731-s001.zip › cancers-2835591-supplementary.pdf]

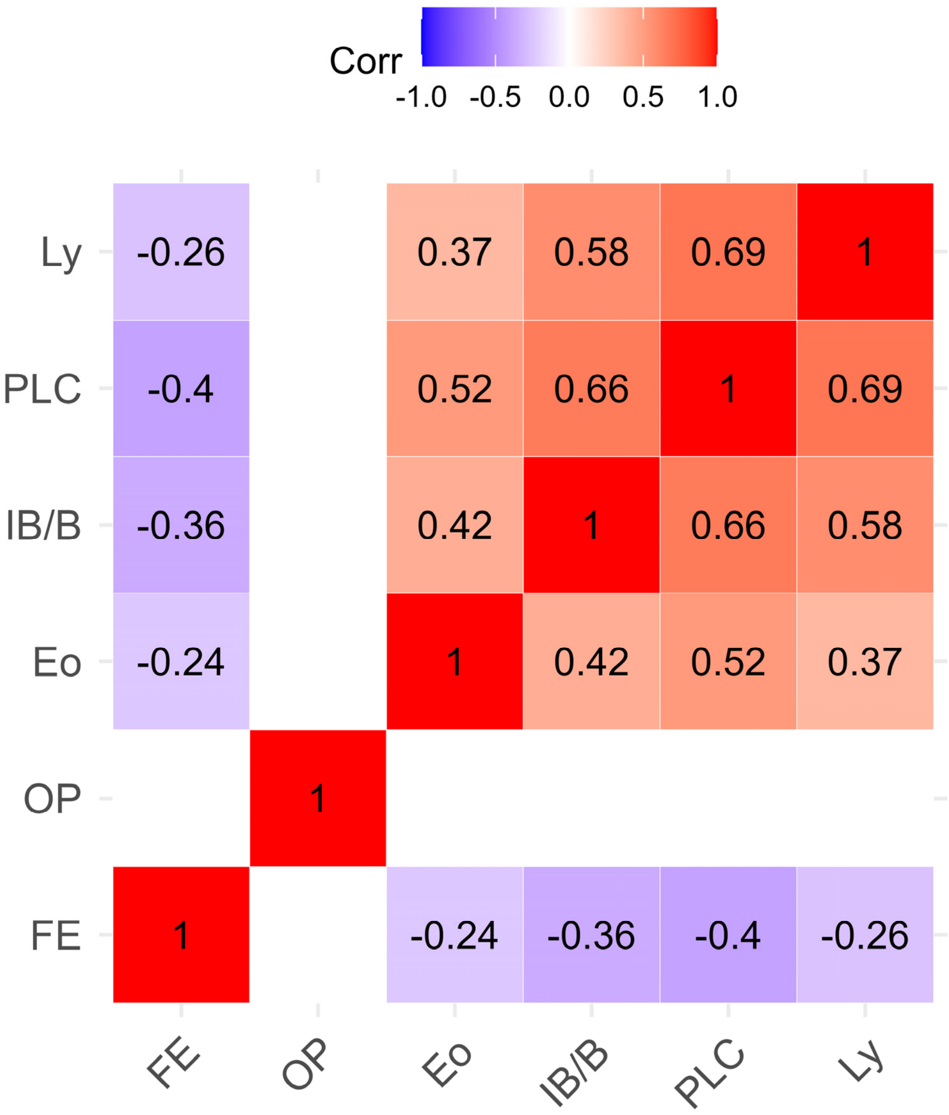

Supplemental Figure S1. Correlation plot of scored features. Correlation analysis by Kendall's tau shows that IB/B, PLC, Eo, Ly, and FE are correlated with each other, while OP is not correlated with any other feature. Numbers printed in each box indecates the Kendall's tau correlation coefficient. Blank boxes indicate pairs that did not reach significance.

ALGORITHM	ACCURACY	AUC	F1	PRECISION	RECALL	TIME
Bagging	0.733	0.722	0.811	0.731	0.913	0.029
Decision Tree	0.731	0.692	0.813	0.724	0.929	0.010
HistGradientBoostingClassifier	0.703	0.709	0.790	0.710	0.895	0.069
XGBoost	0.750	0.751	0.822	0.745	0.920	0.059

Supplemental Table S1. Comparative analysis of evaluated models prior to hyperparameter tuning. Metrics were calculated for each of four selected algorithms to select which model to proceed with hyperparameter tuning. XGBoost had the best performance of the four selected models in all tested areas, except execution time where it performed third best. All values presented as mean of 1000 repeats of random train-test split and model fitting.
AUC, area under the reciever operating curve.

Supplemental Figure S2. Confusion matrices for four evaluated machine learning algorithms. XGBoost performed the best of the four evaluated machine learning algorithms. Confusion matrices taken from a random single repeat of train-test splitting and model fitting.

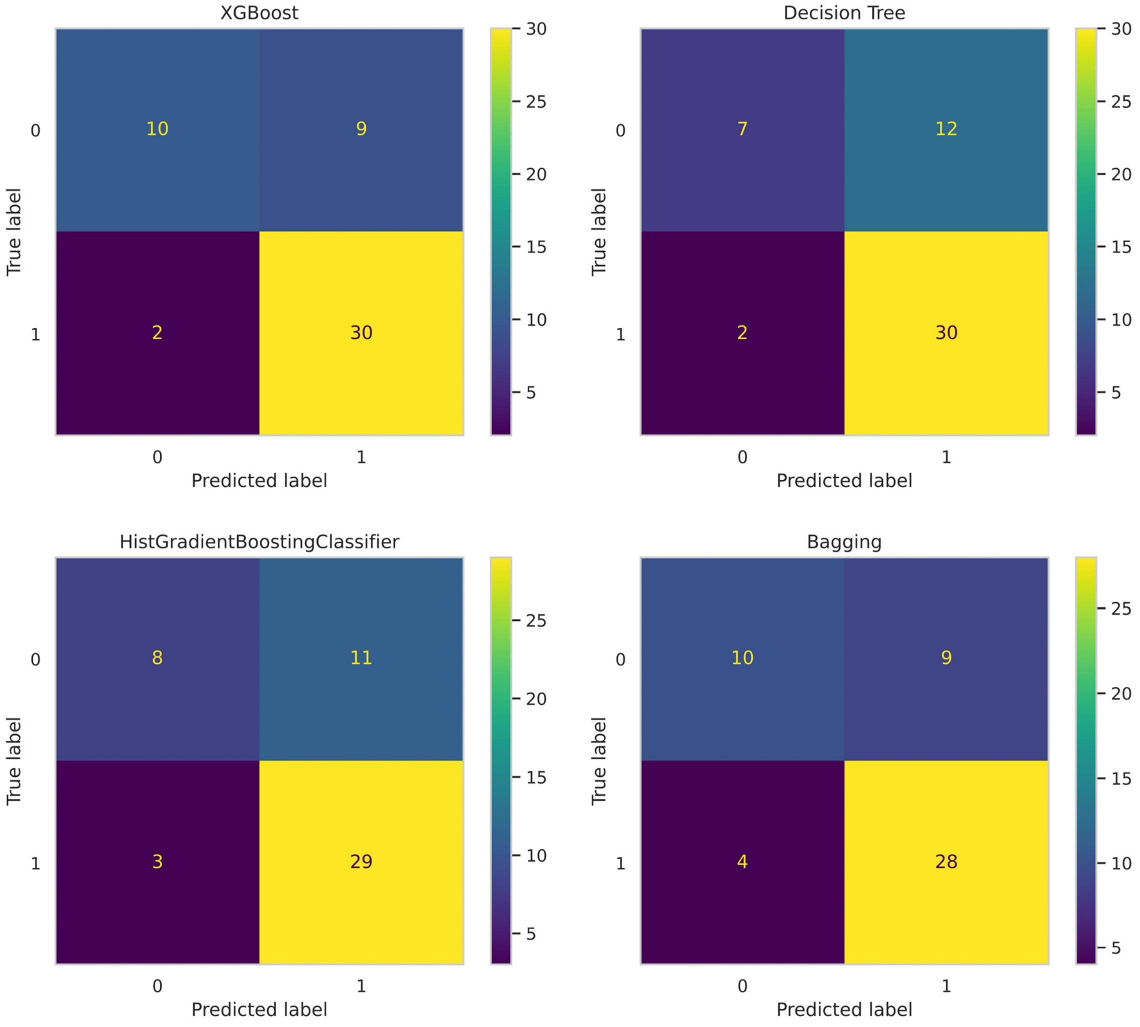

Supplementary Table S2. 10-fold Cross Validation analysis of model performance when removing each scored feature. Performance was most impacted by the removal of IB/B. Certain folds were particularly impacted by the removal of IB/B, FE, or OP.

Accuracy

CV Fold	All	ex-IB/B	ex-PLC	ex-Eo	ex-Ly	ex-FE	ex-OP	Average
**1**	0.80	0.65	0.80	0.80	0.75	0.80	0.80	**0.77**
2	0.80	0.70	0.80	0.80	0.80	0.75	0.75	**0.77**
3	0.80	0.80	0.80	0.80	0.80	0.85	0.75	**0.80**
4	0.80	0.65	0.80	0.75	0.80	0.75	0.80	**0.76**
5	0.70	0.65	0.70	0.70	0.70	0.70	0.65	**0.69**
6	0.85	0.70	0.80	0.85	0.90	0.90	0.90	**0.84**
7	0.85	0.80	0.85	0.85	0.85	0.80	0.85	**0.84**
8	0.80	0.65	0.80	0.80	0.80	0.75	0.80	**0.77**
9	0.70	0.65	0.70	0.70	0.65	0.65	0.70	**0.68**
10	0.70	0.75	0.70	0.70	0.70	0.65	0.60	**0.69**
**Average**	**0.78**	**0.70**	**0.78**	**0.78**	**0.78**	**0.76**	**0.76**	

AUC

CV Fold	All	ex-IB/B	ex-PLC	ex-Eo	ex-Ly	ex-FE	ex-OP	Average
**1**	0.786	0.630	0.813	0.786	0.776	0.807	0.807	**0.772**
2	0.760	0.753	0.787	0.760	0.760	0.627	0.787	**0.748**
3	0.852	0.918	0.857	0.841	0.819	0.841	0.747	**0.839**
4	0.567	0.480	0.567	0.567	0.633	0.493	0.593	**0.557**
5	0.643	0.599	0.632	0.643	0.588	0.714	0.665	**0.641**
6	0.747	0.682	0.778	0.788	0.798	0.919	0.798	**0.787**
7	0.900	0.833	0.900	0.900	0.907	0.747	0.900	**0.870**
8	0.864	0.778	0.864	0.859	0.859	0.768	0.869	**0.837**
9	0.737	0.778	0.768	0.768	0.768	0.742	0.768	**0.761**
10	0.547	0.656	0.536	0.547	0.516	0.589	0.484	**0.554**
**Average**	**0.740**	**0.711**	**0.750**	**0.746**	**0.742**	**0.725**	**0.742**	

F1

CV Fold	All	ex-IB/B	ex-PLC	ex-Eo	ex-Ly	ex-FE	ex-OP	Average
**1**	0.846	0.720	0.846	0.846	0.800	0.846	0.846	**0.822**
2	0.867	0.800	0.867	0.867	0.867	0.839	0.839	**0.849**
3	0.846	0.846	0.846	0.846	0.846	0.880	0.815	**0.847**
4	0.882	0.788	0.882	0.848	0.882	0.848	0.882	**0.859**
5	0.800	0.759	0.800	0.800	0.800	0.800	0.774	**0.790**
6	0.880	0.750	0.846	0.880	0.917	0.917	0.917	**0.872**
7	0.903	0.867	0.903	0.903	0.903	0.875	0.903	**0.894**
8	0.818	0.667	0.818	0.818	0.818	0.783	0.818	**0.791**
9	0.786	0.759	0.786	0.786	0.759	0.759	0.786	**0.774**
10	0.786	0.828	0.786	0.786	0.786	0.741	0.714	**0.775**
**Average**	**0.841**	**0.778**	**0.838**	**0.838**	**0.838**	**0.829**	**0.829**	
